# Genes Whose Gain or Loss-of-Function Increases Endurance Performance in Mice: A Systematic Literature Review

**DOI:** 10.3389/fphys.2019.00262

**Published:** 2019-03-22

**Authors:** Fakhreddin Yaghoob Nezhad, Sander A. J. Verbrugge, Martin Schönfelder, Lore Becker, Martin Hrabě de Angelis, Henning Wackerhage

**Affiliations:** ^1^Exercise Biology Group, Department of Sport and Health Sciences, Technical University of Munich, Munich, Germany; ^2^German Mouse Clinic, Institute of Experimental Genetics, Helmholtz Zentrum München, Neuherberg, Germany; ^3^Chair of Experimental Genetics, School of Life Sciences Weihenstephan, Technical University of Munich, Freising, Germany; ^4^German Center for Diabetes Research, Neuherberg, Germany

**Keywords:** endurance, running, transgenic mice, genetics, mitochondrial biogenesis, GWAS, oxygen uptake, metabolism

## Abstract

Endurance is not only a key factor in many sports but endurance-related variables are also associated with good health and low mortality. Twin and family studies suggest that several endurance-associated traits are ≈50% inherited. However, we still poorly understand what DNA sequence variants contribute to endurance heritability. To address this issue, we conducted a systematic review to identify genes whose experimental loss or gain-of-function increases endurance capacity in mice. We found 31 genes including two isoforms of *Ppargc1a* whose manipulation increases running or swimming endurance performance by up to 1800%. Genes whose gain-of-function increases endurance are *Adcy5, Adcy8*, *Hk2, Il15, Mef2c, Nr4a3, Pck1* (Pepck)*, Ppard, Ppargc1a* (both the a and b isoforms of the protein Pgc-1α), *Ppargc1b, Ppp3ca* (calcineurin)*, Scd1, Slc5a7, Tfe3, Tfeb, Trib3* & *Trpv1*. Genes whose loss-of-function increases endurance in mice are *Actn3, Adrb2, Bdkrb2, Cd47, Crym, Hif1a, Myoz1, Pappa, Pknox1, Pten, Sirt4, Thbs1, Thra, and Tnfsf12*. Of these genes, human DNA sequence variants of *ACTN3*, *ADCY5*, *ADRB2*, *BDKRB2*, *HIF1A*, *PPARD*, *PPARGC1A*, *PPARGC1B*, and *PPP3CA* are also associated with endurance capacity and/or VO_2_max trainability suggesting evolutionary conservation between mice and humans. Bioinformatical analyses show that there are numerous amino acid or copy number-changing DNA variants of endurance genes in humans, suggesting that genetic variation of endurance genes contributes to the variation of human endurance capacity, too. Moreover, several of these genes/proteins change their expression or phosphorylation in skeletal muscle or the heart after endurance exercise, suggesting a role in the adaptation to endurance exercise.

## Introduction

Endurance is a key trait in many sports such as marathon running and triathlon. Endurance is also associated with health as a high endurance capacity is associated with fewer cardiovascular events and reduced all-cause mortality (Kodama et al., 2009). In rats, selection for low endurance capacity is associated with more cardiovascular risk factors than selection for high endurance capacity suggesting a direct link between endurance capacity and disease risk (Wisloff et al., 2005).

Endurance capacity is a multi-factorial trait that depends on several sub-traits and organ systems:

(1) Aerobic capacity (VO_2_max) is influenced by the maximal cardiac output and by the oxygen transport capacity of the blood, and blood volume (Bergh et al., 2000; Lundby et al., 2017).
(2) Skeletal muscle endurance has been linked to type I and II subtype muscle fiber proportions (Costill et al., 1976), muscle capillary density (Brodal et al., 1977), mitochondrial and other metabolic enzyme activities (Gollnick and Saltin, 1983) as well as the glycogen concentration of the exercising muscles (Bergstrom et al., 1967).
(2) Mechanical efficiency describes how much chemical energy is converted into mechanical power (Bassett and Howley, 2000). Efficiency depends on many factors including body weight and height (Maldonado et al., 2002).
(4) Mental endurance depends on the nervous system and is defined as fatigue resistance during prolonged periods of demanding cognitive activity (Van Cutsem et al., 2017).

In relation to human endurance, two important questions are: How much is endurance inherited? What DNA sequence variants affect endurance capacity? Classical genetic studies suggest that maximal aerobic performance variables (i.e., VO_2_max, physical working capacity or threshold values) are between 38 and 94% inherited (Peeters et al., 2009). In the Heritage Study, Bouchard et al. estimated that the VO_2_max was 50% inherited (Bouchard et al., 1998), and that the VO_2_max trainability was 47% inherited (Bouchard et al., 1999). Similarly, the muscle fiber distribution was estimated to be ≈45% inherited (Simoneau and Bouchard, 1995). Collectively, especially the Heritage study data suggest that the variation of major human endurance-related traits depends probably to ≈50% on DNA sequence variation [i.e., genetics, Simoneau and Bouchard, 1995; Bouchard et al., 1998, 1999)] implying that ≈50% is dependent on environmental factors such as endurance training and nutrition.

We still incompletely understand the genetics of human endurance and some researchers are even skeptical about the importance of genetics especially in relation to the VO_2_max. A recent review for endurance-related DNA variants in humans highlighted 93 endurance-associated DNA variants (Ahmetov et al., 2016). Furthermore, a systematic search identified 97 DNA variants that are associated with VO_2_max/peak trainability (Williams et al., 2017). Whilst the effect size of many endurance-associated polymorphisms is small, the effect of a rare *EPOR* DNA variant on the haematocrit and presumably VO_2_max is large, given that one carrier was an Olympic gold medalist in cross country skiing (de la Chapelle et al., 1993). More recently, genome-wide association studies (GWAS) have added to our knowledge of the genetics of human endurance. Here, Rankinen et al. (2016) found in 1520 elite endurance athletes and 2760 controls, no common single nucleotide polymorphism (SNP) profile that distinguishes elite endurance athletes from ethnicity-matched controls (only one SNP near the *GALNTL6* locus was significant across all studies) (Rankinen et al., 2016). Another study in ≈40,000 individuals, and replication in ≈27,000, identified 30 loci that associated with heart rate change at the onset and recovery after exercise. Many of the loci included genes linked to the autonomic nervous system, a known regulator of heart rate (Ramírez et al., 2018). Finally, a study of ≈91,000 individuals identified 14 loci that associated with device-measured physical activity and sleep duration of which several are linked to the central nervous system (Doherty et al., 2018). Given that few physiologically plausible genetic associations have been discovered for endurance, Lundby et al. (2017) question whether “*DNA variants* *in the key physiological pathways for VO_2_max* […] *will be identified for both average individuals and also elite endurance athletes.*” Finally, in his bestselling science book Malcolm Gladwell concludes that 10,000 h is all it takes to achieve expert performance in various fields including sports, leaving little room for genetics or talent (Gladwell, 2008). Thus whilst humans seem to have genetically evolved as an endurance running species (Bramble and Lieberman, 2004), the genetic contribution to the large variation of endurance traits in current human populations is still unclear.

Insights into the genetics of endurance come from studies with good statistical power into the genetics of body height. Body height is not only a ≈80% inherited trait (Silventoinen et al., 2003) but also influences endurance performance, e.g., in rowing, which is an endurance sport (Maldonado et al., 2002). GWAS involving hundreds of thousands of individuals have revealed that the variation of body height is influenced by thousands of SNPs. For example, ≈9,500 SNPs are estimated to explain 29% of human body height variation (Wood et al., 2014). Human body height is additionally influenced by rare, large-effect size mutations of genes such as mutations of *AIP* that cause gigantism (Chahal et al., 2011) or mutations of genes such as *FGFR3* that can cause dwarfism (Foldynova-Trantirkova et al., 2012). Because body height is only one of many endurance performance-limiting factors and already influenced by thousands of DNA variants, it seems likely that even more common DNA sequence variants with small effect sizes and some rarer DNA variants with larger effect sizes combine to explain the effect of DNA sequence variation on human endurance capacity.

The current challenge for molecular exercise physiologists is to start to draw an overall picture of the genetics of human endurance capacity. This picture should both give an idea of the likely number of endurance capacity-influencing DNA variants and identify especially those DNA variants that have a major effect on endurance capacity. A special challenge is to explain the genetics of highly talented elite endurance athletes such as East African runners. Do these athletes share otherwise rare DNA variants with a high effect size as is the case in populations that live at high altitude (Bigham, 2016)? Or do they carry thousands of endurance-promoting DNA variants with small effect sizes? Do the DNA variants mainly affect exons or do they cause variation of regulatory DNA elements?

To identify genes and alleles that can have a major effect on endurance it is especially useful to review the data of transgenic mouse studies (Garton et al., 2016). In transgenic mouse studies, genes are manipulated to produce a gain or loss-of-function of a gene. If this results in a measurable increase of endurance, then the gene is a candidate gene for human endurance capacity, too. The aim of this study was therefore to systematically search the literature for published studies where a gain or loss-of-function mutation of a gene increases endurance capacity in mice. A second aim of the study was to study the identified endurance genes further through bioinformatical analyses.

## Materials and Methods

### Systematic Literature Search

We conducted a systematic review using the PRISMA guidelines (Moher et al., 2009) and included all studies according to the participants, interventions, comparators and outcomes (PICO) process (Schardt et al., 2007). Its main aim was to identify genes whose gain or loss-of-function significantly increases endurance capacity in mice. We first searched the six English-language databases (Google Scholar, Bio Med, Scopus, PubMed, Science Direct, and Web of Science) using our systematic search strategy and used the following combination of search terms: (“mouse” OR “murine” OR “mouse model” OR “mice” OR “mice transgenic”) AND (“overexpression” OR “knock out” OR “knock in” OR “gene transfer techniques” OR “mutagenesis” OR “gene deletion” OR “gene manipulation”) AND [“endurance exercise” OR “swimming” OR “wheel running” OR “endurance capacity” OR “mPXT” (speed progress until exhaustion test in mice) OR “mGXT” (graded maximal exercise in mice)].

### Inclusion and Exclusion Criteria

After eliminating duplicates, we examined the published studies in two stages: First, we reviewed results by title and abstract and then by full-text. At each step, we deleted studies that did not match with the review’s inclusion and exclusion criteria. We included studies in this review if they met the following criteria:

(1) The study needs to be published in a paper or in online peer-reviewed journal;
(2) Publication language must be English;
(3) Each study must show original empirical, primary data/evidence;
(4) Mice must be healthy, and gene manipulation is the only intervention;
(5) Increased endurance capacity must be reported as the distance or time achieved during an endurance exercise test (treadmill running, swimming, wheel-running).
(6) In case an endurance capacity-influencing gene was mentioned more than once, we only analyzed the paper where it was mentioned first.

We excluded studies based on the following criteria:

(1) Rat or *in vitro* study;
(2) No transgenesis, double mutation or long non-coding RNAs manipulation;
(3) Major pathological abnormalities result from the gene manipulation;
(4) Mice are older than 24 months;
(5) No statistically significant effect or no outcome measures;
(6) No wild type mice as controls;
(7) Use of an additional drug treatment or dietary supplement (we included studies where a transgene was induced, e.g., through doxycycline injection).

We illustrate our literature search in Supplementary Data S1.

### Data Extraction

We extracted the following information from each relevant study: author(s), gene name, protein name, method of gene manipulation, acclimated, exercise testing protocols, output measure, endurance capacity for control and transgenic mice, difference between control and transgenic mice as a percentage, age of the mice, mouse strain, additional measurements, and remarks (Supplementary Table S2). Sometimes, the output measure values were presented only in a bar graph and not as a number. In these cases, we manually measured and estimated the relative difference of mean values between controls and transgenic mice by using the bar height. Moreover, we adopted official gene names from the Universal Protein Resource (UniProt, NCBI) and the official gene names may differ from the gene or protein names that are used in the original papers.

### Bioinformatical Analyses

To obtain more information about the identified endurance genes and the proteins that they encode, we asked several research questions and performed bioinformatical analyses to answer these questions. This information is summarized in Supplementary Data S3–S11.

## Results

Initially, we identified 2315 manuscripts with publication dates until January 2018. Based on the title or abstract we excluded 2171 studies. After that, 263 articles remained which we read fully for eligibility. We identified another 43 articles by reviewing the reference lists of the full-text articles or other sources. Finally, we read 144 full-text articles and analyzed 32 articles quantitatively. A PRISMA flowchart of our search and reading strategy is in the Supplementary Data S1. We used the information of this systematic literature search and several bioinformatical analyses to answer several research questions which are stated as headers below.

### The Gain or Loss-of-Function of What Genes Increases Endurance Performance?

**FIGURE 1:**
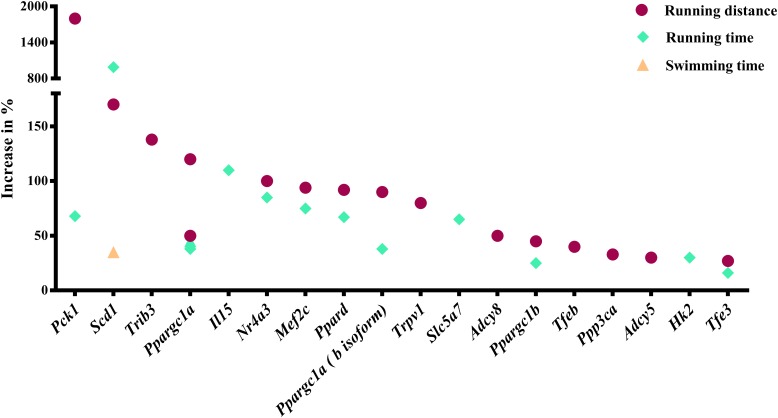
Genes whose gain-of-function increases running or swimming endurance in mice. Percentage increase in % were calculated by direct comparison the control animals. Finally, genes are plotted from high to low effect size.

**FIGURE 2:**
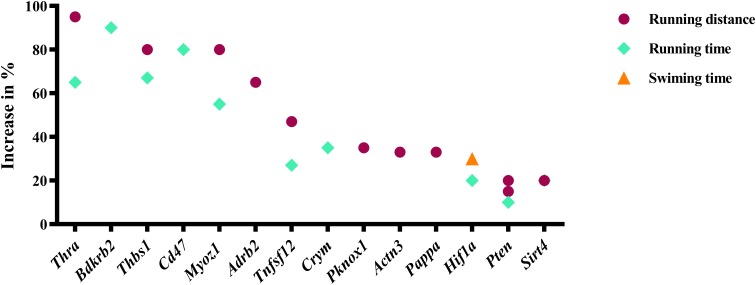
Genes whose loss-of-function increases running or swimming endurance in mice. Percentage increase in % were calculated by direct comparison the control animals. Finally, genes are plotted from high to low effect size.

Our analysis revealed 31 genes/isoforms including two isoforms of *Ppargc1a* whose gain or loss-of-function increased endurance performance in mice. Specifically, we identified 19 genes [*Adcy5, Adcy8*, *Hk2, Il15, Mef2c, Nr4a3, Pck1* (Pepck), *Ppard, Ppargc1a* (both the a and b isoforms of the mitochondrial biogenesis regulator Pgc-1α), *Ppargc1b, Ppp3ca* (calcineurin)*, Scd1, Slc5a7, Tfe3, Tfeb, Trib3, and Trpv1;* Figure 1] whose gain-of-function increased endurance capacity in mice. We also found 14 genes (*Actn3, Adrb2, Bdkrb2, Cd47, Crym, Hif1a, Myoz1, Pappa, Pknox1, Pten, Sirt4, Thbs1, Thra*, *Tnfsf12*; Figure 2) whose loss-of-function increases endurance capacity. Collectively, we will refer to these genes as endurance genes for simplicity. The relative increase ranged from 12% for *Pten* to 1800% for *Pck1* (Figure 1,2). To explain, if the mean value for the wildtype mouse was e.g., 100 units in an endurance test, then the transgenic mice achieved on average between 112 units (i.e., an increase of 12 units or %) or 1900 units (i.e., an increase by 1800 units or %), respectively. The detailed experimental design and endurance performance data for each transgenic and wildtype mouse pair are summarized in Table 1 and in full detail in Supplementary Data S2.

**Table 1:** Genes whose transgenesis in mice increases endurance capacity.

Gene symbol	Protein name	Transgenesis	Output measure	Increase (%)	Reference
*Pck1*	Phosphoenolpyruvate carboxykinase 1, cytosolic	Conditional overexpression	Time to exhaustion Distance	+68% +1800%*	Hakimi et al., 2007
*Scd1*	Stearoyl-Coenzyme A desaturase 1	Conditional overexpression	Time to exhaustion (run) Time to exhaustion (swim) Distance	+989% +35% +170%	Rogowski et al., 2013
*Trib3*	Tribbles pseudokinase 3	Conditional overexpression	Total work	+138%	An et al., 2014
*Ppargc1a*	Peroxisome proliferative activated receptor, gamma, coactivator 1 alpha	Conditional overexpression	Time to exhaustion #1 Distance Max speed	+38–44.9% +50–120%* +24–34%	Calvo et al., 2008
*Il15*	Interleukin 15	Conditional overexpression	Time to exhaustion	+110%*	Quinn et al., 2013
*Nr4a3*	Nuclear receptor subfamily 4 group A member 3	Conditional overexpression	Time to exhaustion Distance	+85%* +100%*	Pearen et al., 2012
*Ppargc1a (b isoform)*	Peroxisome proliferative activated receptor, gamma, coactivator 1 alpha	Conditional overexpression	Time to exhaustion Distance Max speed	+36–40% +83-100% +35-40%	Tadaishi et al., 2011
*Thra*	Thyroid hormone receptor alpha (mitochondrial T3 receptor)	Global knock out	Time to exhaustion Distance	+65* +95%*	Pessemesse et al., 2012
*Mef2c*	Myocyte enhancer factor 2C	Gain-of-function	Time to exhaustion Distance	+75% +94%	Potthoff et al., 2007
*Ppard*	Peroxisome proliferator activated receptor delta	Conditional overexpression	Time to exhaustion Distance	+67% +92%	Wang et al., 2004
*Bdkrb2*	Bradykinin receptor, beta 2	Global knock out	Time to exhaustion	+90%*	Reis et al., 2015
*Thbs1*	Thrombospondin 1	Global knock out	Time to exhaustion Distance Max speed	+67% +80% +11%	Malek and Olfert, 2009
*Cd47*	CD47 antigen (Rh-related antigen, integrin-associated signal transducer)	Global knock out	Time to exhaustion	+80%*	Frazier et al., 2011
*Myoz1*	Myozenin 1	Global knock out	Time to exhaustion Distance	+55%* +75%*	Frey et al., 2008
*Trpv1*	Ransient receptor potential cation channel, subfamily V, member 1	Global overexpression	Distance	+80%*	Luo et al., 2012
*Slc5a7*	Solute carrier family 5 (choline transporter), member 7	Global overexpression	Time to exhaustion Max speed	+ 65% + 10%*	Holmstrand et al., 2014
*Adrb2*	Adrenoceptor beta 2	Global knock out	Distance	+65%*	Voltarelli et al., 2012
*Adcy8*	Adenylate cyclase 8	Conditional overexpression	Distance	+50%*	Esposito et al., 2008
*Tnfsf12*	Tumor necrosis factor (ligand) superfamily, member 12	Global knock out	Time to exhaustion Distance	+27% +47%	Sato et al., 2013
*Ppargc1b*	Peroxisome proliferative activated receptor, gamma, coactivator 1 beta	Conditional overexpression	Time to exhaustion Distance	+25% +45%	Arany et al., 2007
*Tfeb*	Transcription factor EB	Inducible conditional overexpression	Distance	+40%*	Mansueto et al., 2017
*Crym*	Crystallin, mu	Global knock out	Time to exhaustion	+35%*	Seko et al., 2016
*Pknox1*	Pbx/knotted 1 homeobox	Conditional knock out	Distance	+35%	Kanzleiter et al., 2014
*Actn3*	Actinin alpha 3	Global knock out	Distance	+33%	MacArthur et al., 2007
*Ppp3ca*	Calcineurin; protein phosphatase 3, catalytic subunit, alpha isoform	Conditional overexpression	Distance	+33%	Jiang et al., 2010
*Pappa*	Pregnancy-associated plasma protein A; pappalysin 1	Global knock out	Distance (median)	+33%	Conover et al., 2016
*Hif1a*	Hypoxia inducible factor 1 subunit alpha	Conditional knock out	Time to exhaustion (swim) Time to exhaustion (uphill)	+30%* +20%*	Mason et al., 2004
*Adcy5*	Adenylate cyclase 5	Conditional overexpression	Distance	+30%*	Esposito et al., 2008
*Hk2*	Hexokinase 2	Conditional overexpression	Time to exhaustion	+30%*	Fueger et al., 2005
*Tfe3*	Transcription factor to IGHM enhancer 3 (transcription factor E3)	Conditional overexpression	Time to exhaustion Distance	+16% +27%	Iwasaki et al., 2012
*Pten*	Phosphatase and tensin homolog	Conditional knock out	Time to exhaustion Distance	+10% +15–20%	Yue et al., 2016
*Sirt4*	Sirtuin 4	Global knock out	Time to exhaustion Distance	+20% +20%	Laurent et al., 2013


### Do Endurance Genes Overlap With Human Genes Where DNA Variants Are Associated With Human Endurance, VO_2_max Trainability and Other Endurance-Related Traits?

To study whether endurance genes also play a role in the variation of human endurance, we overlapped our set of endurance genes with a list of human genes where DNA variants associate with endurance (Ahmetov et al., 2016) and genes where DNA variants associate with VO_2_max trainability (Williams et al., 2017). Moreover, we searched for phenotypes for endurance genes reported in human GWAS by searching the human GWAS catalog. The first overlap analysis we screened the human homologs of the mouse genes. Here, we revealed that human endurance gene variants of *ACTN3*, *ADRB2*, *BDKRB2*, *HIF1A*, *PPARD*, *PPARGC1A*, *PPARGC1B*, and *PPP3CA* are also associated with human endurance (Ahmetov et al., 2016). Moreover, DNA variants linked to *ADCY5*, *PPARD*, and *HIF1A* are associated with VO_2_max trainability in humans (Williams et al., 2017). In a second step, we investigated whether the identified endurance genes were linked in GWAS to phenotypes that are potentially relevant for endurance performance. To do so, we performed a GWAS catalog search for each gene. This search revealed several associations between endurance genes and a several physiological and pathological human phenotypes (Supplementary Data S3). Associations of potential relevance for endurance performance include an association of *PCK1* with the hemoglobin concentration (Astle et al., 2016), an association of *PPARGC1A* with the resting heart rate (Eppinga et al., 2016), an association of *SCD* with metabolic traits (Suhre et al., 2011) specifically blood levels of myristate (14:0)/myristoleate (14:1n5) (Shin et al., 2014), and an association of *TFEB* with left ventricular wall thickness (Wild et al., 2017). Collectively, these analyses demonstrate that some endurance genes are assocuated with human endurance-associated traits, too.

### How Much Does the DNA Sequence of Human Endurance Gene Exomes Vary in 60,706 Humans?

Next we used the ExAC browser to explore the extent to which the DNA sequence of human endurance gene exoms varies (Lek et al., 2016). This analysis revealed extensive genetic variation of endurance genes in humans. On average, each human homologue of an endurance gene had 174 missense DNA variants, 5 loss-of-function DNA variants and 11 copy number DNA variants. Additionally, for *ACTN3, CRYM, TFE3*, and *THRA* homozygous loss-of-function DNA variants were reported. For *ACTN*3 it is already known that a loss-of-function can be tolerated as ≈20% of the population are homozygous for a ACTN3 R577X variant (Yang et al., 2003). This and the results of GWAS s suggest a large amount of common or rare, functionally relevant DNA sequence variation in the human homologues of mouse endurance genes.

### In What Human Tissues Are Endurance Genes Expressed?

**FIGURE 3:**
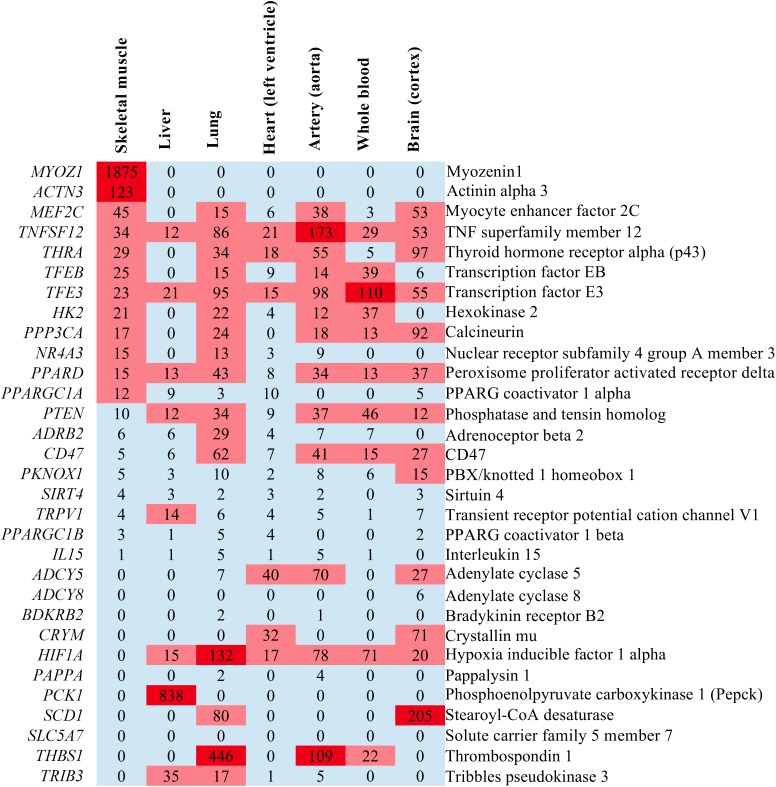
Heatmap illustrating the expression levels of endurance genes in transcripts per million (TPM) in some endurance exercise organs. If no expression value was given in GTEx Portal, we recorded the expression as “0” which signifies either no expression or no data.

We have already mentioned that endurance is a trait that is determined by the function and interplay of several organ systems. These include skeletal muscle as the key force-generating and energy converting organ, the liver for glycogen storage and gluconeogenesis, the oxygen-delivering organs lung, heart, vasculature and blood as well as the brain due to its role in mental fatigue. To study the expression in resting human organs, we retrieved gene expression data from the GTEx Portal database (GTEx Consortium, 2015) and plotted this as a heat map (Figure 3 and Supplementary Data S5). This reveals that *Myoz1* and *Actn3* are selectively expressed in skeletal muscle whereas *Pck1* is selectively expressed in the liver, at least at rest. In addition, several other genes, such as *Sirt4, Ppargc1a, Il15Adcy8Bdkrb2, Pappa*, and *Slc5a7*, are not expressed in these selected organs.

### Do Endurance Genes Change Their Expression in Muscle After Human Endurance and Resistance (Strength) Exercise? What Endurance Genes/Proteins Are Detected as Phosphorylated Proteins at Rest or After High Intensity Exercise in Human Muscle?

**FIGURE 4:**
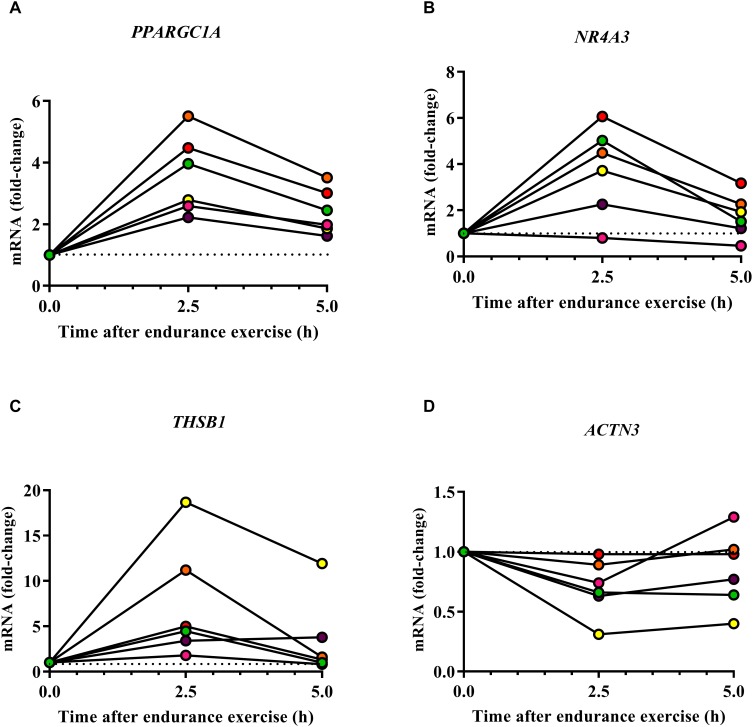
Effect of human endurance exercise on the expression of *PPARGC1A* **(A)**, *NR4A3* **(B)**, *THSB1* **(C)**, and *ACTN3* **(D)** in the vastus lateralis 2.5 and 5 h after exercise (Vissing and Schjerling, 2014).

Several genes may be expressed at low levels in resting human organs but may increase their expression in response to endurance exercise. One example is the gene *Ppargc1a* which encodes various isoforms of the mitochondrial biogenesis regulator Pgc-1α. The expression of *Ppargc1a* increases in response to endurance exercise both in mouse (Baar et al., 2002) and human skeletal muscle (Pilegaard et al., 2003). Moreover, proteins encoded by endurance genes may become phosphorylated after a bout of endurance exercise as can be demonstrated by phosphoproteomics (Hoffman et al., 2015). To test whether endurance genes/proteins change their expression or phosphorylation after a bout of endurance exercise, we re-analyzed published datasets (Vissing and Schjerling, 2014; Hoffman et al., 2015). These analyses reveal that *PPARGC1A* (encoding PGC-1α), *NR4A3* which encodes a nuclear hormone receptor and *THSB1*, which encodes thrombospondin 1, are examples for genes that increase their expression 2.5 and 5 h in the vastus lateralis after cycling for 120 min at 60% of the VO_2_peak (Figure 4 and Supplementary Data S6; Vissing and Schjerling, 2014). In contrast, *ACTN3* decreases its expression after human endurance exercise (Figure 4). Whilst the direction of the expression changes of *PPARGC1A*, *NR4A3*, and *ACTN3* is consistent with the respective mouse phenotypes, *THSB1* expression increases after endurance exercise but a global deletion of *Thsp1* increases capillarity and exercise capacity in mice (Malek and Olfert, 2009). Thus, *THSB1* expression after endurance exercise suggests that it promotes a reduced adaptation to endurance exercise.

In addition, proteins encoded by *ACTN3, ADRB2, MEF2C* and *TFEB* were detected as phosphorylated proteins in vastus lateralis samples after a single bout of high-intensity cycle exercise at ≈90% of the maximal power output (Wmax) for ≈10 min (Supplementary Data S7). Of these proteins, TFEB phosphorylation significantly decreased from pre to post exercise (Hoffman et al., 2015). Collectively these expression and phosphoproteomics data show that some endurance genes change their expression or phosphorylation in human skeletal muscle after exercise and are therefore potential regulators of skeletal muscle endurance adaptations.

As we have mentioned, skeletal muscle is not the only organ whose function limits endurance performance. The heart is a particularly important endurance organ. Endurance training induces the athlete’s heart or physiological cardiac hypertrophy. Such a heart has a hypertrophied left ventricle, which increases stroke volume, cardiac output and the VO_2_max (Ekblom, 1968; Ellison et al., 2012). To test whether endurance genes change their expression in a mouse model of physiological cardiac hypertrophy induced through 8 weeks of swimming versus a pathologically hypertrophied heart achieved through isoproterenol treatment versus sedentary controls, we re-analyzed the dataset of Galindo et al. (2009). This analysis revealed that depending on the measured probe, *Ppargc1a* increased by 4.27 and 6.96-fold, whereas *Thra* decreased -2.50-fold, *Ppard* by -2.86-fold and *Tnfsf12* by -1.80 fold, in the physiologically hypertrophied heart specifically when compared to sedentary control, respectively (Supplementary Data S8). Some endurance genes alter their expression in response to both physiological and pathological hypertrophy of the heart. *Hif1a* increases by 1.99-fold, *Mef2c* by 1.99 – 2.90-fold and *Pten* by 2.05-fold in during physiological hypertrophy, while *Hif1a* decreases -1.87-fold, *Mef2c*-2.16-fold, and *Pten* by -1.74 -1.84-fold during pathological hypertrophy. *Ppargc1b* and *Hk2* specifically decrease their expression in pathological hypertrophy by -2.80-fold, and -1.95-fold, respectively, and not during physiological hypertrophy. These data demonstrate that endurance exercise can induce or repress the expression of some endurance genes in the heart of mice.

### Do Endurance Genes Interact With Each Other? Do Endurance Genes Share Common Features?

**FIGURE 5:**
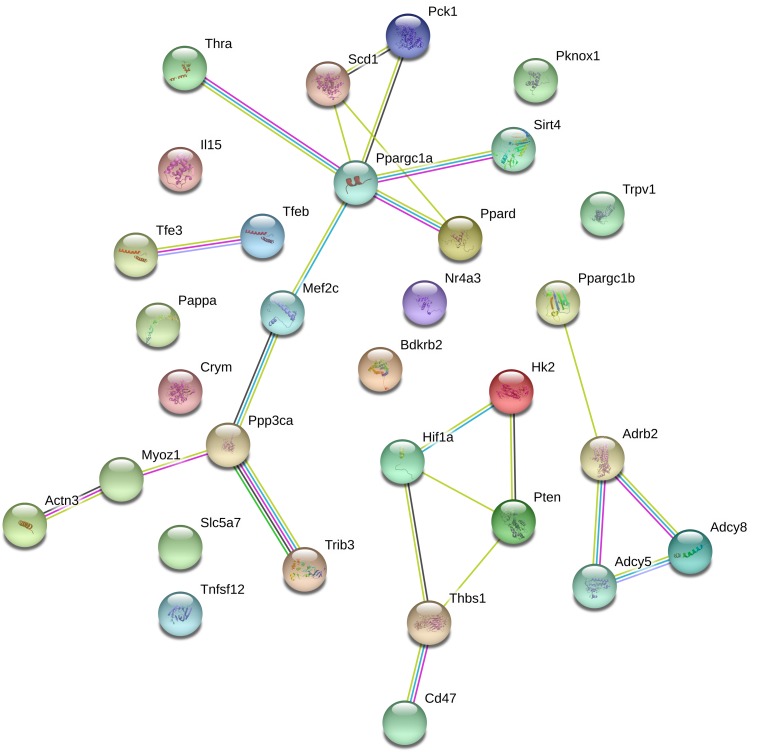
String analysis for endurance genes. Lines between proteins indicate evidence of association.

Next, we investigated whether endurance genes are functionally linked and whether these genes are enriched among specific classes of genes such as genes that share a common domain or molecular function. First, we performed a STRING analysis that predicts direct physical and other associations for a group of proteins (Supplementary Data S9; Szklarczyk et al., 2017). Figure 5 illustrates the results of this analysis. Clusters in this figure are linked to the mitochondrial biogenesis regulator Pgc1a (encoded by *Ppargc1a*), the calcium/calmodulin-stimulated phosphatase calcineurin encoded by *PPP3CA* and the β2-adrenoceptor encoded by *Adrb2*. This suggests some functional interaction between endurance genes.

We then performed a functional enrichment analysis to see whether the endurance performance-increasing genes share common features. It has been pointed out that such enrichment analyses may be erroneous if the enrichment is not adjusted to the transcriptome of the tissue of interest (Timmons et al., 2015). However, the endurance effect of our gene list could be due to expression in several tissues and so we are unable to use one tissue-specific background list. Instead, we have conducted a general meta-enrichment analysis using ToppGene (Chen et al., 2009). This analysis has revealed potentially relevant enrichments that include the mouse phenotype “abnormal heart rate” (MP:0001629) for 8 of the genes and “abnormal metabolism” (MP:0005266) for 13 of the endurance genes. All significant enrichments are listed in Supplementary Data S10.

### How Many Endurance Genes/Proteins Are Predicted to Be Secreted?

Finally, ≈3000 proteins are predicted to be secreted from cells. To find out how many endurance genes are predicted to encode secreted proteins, we retrieved the list of secreted genes/proteins from Protein Atlas^1^ and compared these genes with the list of endurance genes. We identified three endurance genes, *Pappa (*Pappalysin 1), *Thbs1* (Thrombospondin 1) and *Tnfsf12* (TNF superfamily member 12) that encode proteins that are predicted to be secreted (Supplementary Data S11). Together, these secreted proteins could play possible roles in inter-organ signaling in relation to acute exercise or adaptation to chronic exercise.

## Discussion

The main finding of this systematic review is the identification of 31 genes whose gain or loss-of-function increases endurance performance in mice by up to 1800% when compared to wildtype control mice. Further bioinformatical analyses reveal the DNA sequence variability of these genes in humans, their organ-specific expression pattern, functional links in-between these genes and the proteins they encode, and a role for some endurance genes during adaptation to endurance exercise. This endurance gene list also provides an up-to-date candidate list for more targeted human genetic analyses for endurance performance or trainability.

### Relevance of the Identified Endurance Genes for Explaining Human Endurance

A key question is whether the mouse endurance genes are relevant for the genetics of human endurance. Our analyses suggest that this is probably the case. First, human variants of *ACTN3*, *ADRB2*, *BDKRB2*, *HIF1A*, *PPARD*, *PPARGC1A*, *PPARGC1B*, and *PPP3CA* are associated with human endurance (Ahmetov et al., 2016) and human variants of *ADCY5*, *PPARD* and *HIF1A* are associated with VO_2_max trainability (Williams et al., 2017). Second, GWAS studies identified SNPs linked to the human homologues of endurance genes to the hemoglobin concentration (Astle et al., 2016), resting heart rate (Eppinga et al., 2016), metabolic traits (Suhre et al., 2011; Shin et al., 2014), and left ventricular wall thickness (Wild et al., 2017). Finally, we found that the exon DNA sequence of human endurance genes varies considerably. On average, each endurance gene has 174 missense DNA variants, 5 loss-of-function DNA variants and 11 copy number DNA variants in 60,706 individuals (Lek et al., 2016; note that this data refers to the number of alleles and that the number of carriers is much higher). However, associations or DNA variants of endurance genes does not mean that they actually increase endurance in humans (Houweling et al., 2018). Associations need to be replicated and supported by functional analysis, such as in mouse models, to find mechanisms responsible for endurance phenotypes (Eynon et al., 2017). So far, only for *ACTN3* there is consistent data showing that a common DNA variant in humans influences muscle performance, which is similar in the *ACTN3* mouse model (Houweling et al., 2018). Collectively, this suggests that variants of the human homologues of mouse endurance genes could contribute to the variation of endurance-related traits seen in the human population. This has to be replicated in future studies.

### Several Endurance Genes Affect Mitochondrial Biogenesis and Energy Metabolism

The majority of the endurance genes identified in our study are linked to skeletal muscle metabolism and mitochondrial biogenesis. Here, the transcriptional co-factor Pgc-1α plays a key role (Lin et al., 2002). The authors of the original *Ppargc1a* (which encodes Pgc-1α) overexpression study did not test the endurance capacity of the transgenic mice. However, subsequent studies demonstrated the effect of the overexpression of *Ppargc1a* (Calvo et al., 2008), of the b-isoform of *Ppargc1a* (Tadaishi et al., 2011) and of *Ppargc1b* (Arany et al., 2007) on endurance capacity, skeletal muscle mitochondrial biogenesis and muscle fiber-related gene expression. A related factor is *Ppard* whose overexpression has similar effects on mitochondrial biogenesis, muscle fiber-related gene expression and endurance capacity (Wang et al., 2004). Many of the other endurance genes regulate the expression of *Ppargc1a* isoforms or of *Ppard* or the activity of the proteins that these genes encode which explains their effect on endurance capacity. *Ppargc1a* expression also increases after endurance exercise in mouse (Baar et al., 2002) and human skeletal muscle (Pilegaard et al., 2003; Figure 4) as well as the heart during swimming-induced cardiac hypertrophy (Supplementary Data S8) suggesting that it is a mediator of muscle and heart adaptations to endurance exercise (Holloszy, 1967).

Two of the endurance genes haven been linked to thyroid hormone signaling. They are *Crym*, which encodes a thyroid hormone-binding crystallin (Seko et al., 2016) and *Thra* which encodes a nuclear thyroid hormone receptor (Pessemesse et al., 2012). The mechanisms are probably linked to the effect of thyroid hormones on mitochondrial biogenesis via Pgc-1α and related factors (Weitzel and Alexander Iwen, 2011). Interestingly, some endurance athletes have been reported to take thyroid medication as a treatment (Hart, 2017) which is a concern as the real purpose might be to enhance endurance capacity through thyroid hormone treatment.

Pgc-1α and related factors are, however, not the only regulators of mitochondrial biogenesis, muscle metabolism and fiber type-specific gene expression. A different group includes *Ppp3ca* which encodes a subunit the Ca^2+^-activated phosphatase calcineurin (Jiang et al., 2010), the calcineurin regulator calsarcin-2 encoded by *Myoz1* (Frey et al., 2008) and the calcineurin-regulated transcription factor Tfeb which promotes mitochondrial biogenesis and other metabolic adaptations (Mansueto et al., 2017). A genome-wide association study has also linked *TFEB* to left ventricular wall thickness and TFEB phosphorylation decreased significantly from pre to post exercise (Hoffman et al., 2015), suggesting that TFEB may regulate skeletal muscle and heart adaptations to endurance exercise.

### Some Endurance Genes Have an Effect on the Oxygen-Delivery System

In humans, a key determinant of a high endurance capacity is the VO_2_max, which depends on the maximal oxygen transport capacity. This in turn depends on the maximal cardiac output, which is increased in the athlete’s heart, and on the oxygen transport capacity of the blood (Bergh et al., 2000; Lundby et al., 2017). Earlier studies reported that cardiac-specific expression of the kinase Mek1 increased cardiac function (Bueno et al., 2000) and that the expression of a dominant negative form of Pi3k in the heart prevented physiological cardiac hypertrophy (i.e., the development of an athlete’s heart) after swimming in mice (McMullen et al., 2003). Unfortunately, whether the heart-specific overexpression of these two genes increased exercise capacity in mice was not tested. In another study, researchers overexpressed the catecholamine-related, adenylyl cyclase-encoding genes *Adcy5* and *Adcy8* in the heart. They found that this overexpression increased cardiac contractility and endurance capacity in the transgenic mice when compared to wildtype controls (Esposito et al., 2008). Also, *ADCY5* gene variants are associated with VO_2_max trainability in humans (Williams et al., 2017) and for *ADCY5* and *ADCY8* together 659 different missense DNA variants, 17 loss-of-function DNA variants and 13 DNA copy number variants have been reported for 60,706 humans in the Exac study (Lek et al., 2016). Collectively, this suggests that numerous DNA variants of *ADCY5* and *ADCY8* might contribute to the variation of VO_2_max trainability and perhaps VO_2_max seen in humans. In a different model, the knockout of *Thbs1* (encoding thrombospondin-1) increased skeletal muscle and cardiac capillary density, left ventricular size and endurance capacity (Malek and Olfert, 2009). Together this demonstrates changed activity of some endurance genes may contribute to the development of an Athlete’s heart.

Other endurance genes change their expression in the heart in endurance-exercising mice (Figure 3; especially *Ppargc1a* appears to increase during physiological cardiac hypertrophy) or are associated with cardiac phenotypes in GWAS studies (Supplementary Data S3). Here, the SNP near *PCK1* was associated with the hemoglobin concentration (Astle et al., 2016). However, it is unclear whether the overexpression of *Pck1* (encoding Pepck) in skeletal muscle can explain an increased hemoglobin concentration (Hakimi et al., 2007).

### Endurance Genes, Neural and Behavioral Mechanisms

Mental fatigue has recently been highlighted as an endurance-influencing factor (Van Cutsem et al., 2017) but we know little about the molecular mechanisms that influence mental fatigue, neural function and behavior in relation to endurance. Two of the endurance genes are linked to the nervous system. Acetylcholine is synthesized from acetyl-CoA and choline and released from motor endplates to cause muscle fibers to contract. Interestingly, the overexpression of the sodium-choline channel gene *Slc5a7* increased choline transport, endurance capacity but not strength and physical activity in mice (Holmstrand et al., 2014). The overexpression of *Pck1* in skeletal muscle not only increased the endurance capacity most (Figure 1) but made these mice hyperactive in their home cages (Hakimi et al., 2007). How the high expression of a gluconeogenetic enzyme in skeletal muscle can increase spontaneous activity in mice is unclear.

### Limitations

Like in our earlier review on hypertrophy-causing genes (Verbrugge et al., 2018), a limitation of this review is that the manipulated genes are subjectively chosen by the researchers of each study. Moreover, many researchers do not test whether a manipulated gene changes endurance exercise capacity. The consequence is that the list of manipulated genes and the mice that are tested in an endurance test is subjective, resulting in a biased list of endurance genes. Currently, the International Mouse Phenotyping Consortium (IMPC) generates and phenotypically analyses 20,000 mouse lines^2^ but measurement of endurance capacity is not included in the first-line analysis (Brown and Moore, 2012). Here a triage system might be useful, so that mice that have increased cardiac function, a higher haematocrit or other endurance-associated phenotypes are then also tested in an endurance test.

Another limitation of the study is that the variation of endurance performance also depends on the endurance tests used in the individual studies. For example, if the endurance performance of the same wildtype and transgenic animals is measured in a graded exercise test versus a time trial test then the results will differ. For example, in the *Pck1* mouse study the researchers found that wildtype mice ran ≈200 m at a speed of 20 m/min whereas *Pck1*-overexpressing mice ran in-between 2000 and 6000 m, which is on average ≈1800% more than the wildtype mice. In contrast, when the same mice were compared in a graded exercise test with an increase of 1 m/min every minute then the *Pck1*-overexpressing mice achieved a maximum speed of ≈50 m/min whereas the wildtype controls ran up to a speed of ≈20 m/min. This is a much smaller increase of 150% (Hakimi et al., 2007). Together this demonstrates that the type of endurance performance test can greatly affect the outcome and differences in percent between wildtype and transgenic mouse strains. Generally, researchers should aim for standardized protocols for running tests to exhaustion or for graded exercise tests. Booth et al. give some specific recommendations for such exercise tests in mice (Booth et al., 2010).

A final limitation of this study is that we do not include mouse models where the gain or loss-of-function of a gene reduces endurance performance. Such mouse models can provide important insights into genes that influence endurance performance (Garton et al., 2016). However, the major problem with such mouse models is that it is difficult to judge whether the decrease in performance is because of the reduction of a true endurance-increasing variable or whether endurance is decreased because of a disease. For example, we would expect that almost all tumor-bearing mice have a reduced endurance capacity even though the effect of the genetic manipulation causes tumor and does not directly affect endurance-influencing variables.

## Summary and Conclusion

A high endurance capacity is important for many sports, is associated with good health, low mortality and many endurance-associated traits are ≈50% inherited. However, how DNA variants contribute to the variation of human endurance and especially of VO_2_max and VO_2_max trainability is still incompletely understood and some aspects are controversial (Lundby et al., 2017). The contribution of this study to our understanding of endurance genetics is a list of 31 genes whose gain or loss-of-function increases endurance performance by up to 1800% in mice. Many of the identified endurance genes are linked to biological pathways that are relevant for endurance, especially mitochondrial biogenesis and muscle metabolism. Moreover, exome sequencing data for 60,706 individuals contain a large number of amino acid sequence and/or function-changing DNA variants for these genes (Lek et al., 2016), suggesting that human variants of these genes partially explain the variation of endurance capacity. In contrast, few endurance genes are linked to the oxygen-transporting systems that limit the VO_2_max. Here the best VO_2_max-influencing candidate genes are the *ADCY5* and *ADCY8* adenylyl cyclase genes that increase cardiac contractility (Esposito et al., 2008). *ADCY5* gene variants are also associated with VO_2_max trainability in humans (Williams et al., 2017) and more than 600 types of function-altering DNA variants have been reported for *ADCY5* and *ADCY8* in 60,706 humans (Lek et al., 2016). Still, this leaves much of the known genetic variability of the VO_2_max and VO_2_max trainability unexplained. Practically, this endurance gene list and our earlier list of hypertrophy-promoting list may be useful for more targeted and in depth genetic analyses of elite endurance athletes such as East African runners.

## Author Contributions

FYN and SV conducted the systematic paper analysis. FYN, SV, MS, and HW did the bioinformatical analyses. LB and MH revised the manuscript. HW drafted the manuscript and all authors contributed to writing the manuscript.

## Conflict of Interest Statement

The authors declare that the research was conducted in the absence of any commercial or financial relationships that could be construed as a potential conflict of interest.

## Footnotes

1 https://www.proteinatlas.org/humanproteome/secretome

2 http://www.mousephenotype.org/
